# Ecological aspects of the Phlebotominae fauna (Diptera: Psychodidae) in the Xakriabá Indigenous Reserve, Brazil

**DOI:** 10.1186/1756-3305-7-220

**Published:** 2014-05-12

**Authors:** Felipe Dutra Rêgo, Paloma Helena Fernandes Shimabukuro, Patrícia Flávia Quaresma, Igor Rismo Coelho, Gabriel Barbosa Tonelli, Kelly Medrado Scofield Silva, Ricardo Andrade Barata, Edelberto Santos Dias, Célia Maria Ferreira Gontijo

**Affiliations:** 1 Grupo de Estudos em Leishmanioses, Centro de Pesquisas René Rachou, Fundação Oswaldo Cruz, Av. Augusto de Lima, 1715 Barro Preto, CEP 30190-002 Belo Horizonte, Minas Gerais, Brazil; 2 Laboratório de Ecologia e Sistemática de Abelhas, Sala 245, Bloco E2 Instituto de Ciências Biológicas, Universidade Federal de Minas Gerais Avenida Presidente Antônio Carlos, 6627 Caixa Postal 486, CEP 31270-901 Belo Horizonte, Minas Gerais, Brazil; 3 Departamento de Ciências Biológicas, Universidade Federal dos Vales do Jequitinhonha e Mucuri - Campus JK, BR 367, Alto da Jacuba, CEP 39100-000 Diamantina, MG, Brazil

**Keywords:** Phlebotominae fauna, Ecological aspects, Minas Gerais State, *Nyssomyia*, *Lutzomyia*, *Martinsmyia*, *Leishmania*

## Abstract

**Background:**

Sand fly collections were performed to study ecological aspects of the Phlebotominae fauna of the Xakriabá Indigenous Reserve, an area with endemic cutaneous leishmaniasis, located in the state of Minas Gerais, Brazil.

**Methods:**

The collections were performed in peridomicile areas and along trails previously selected for the study of wild and synanthropic *Leishmania* hosts. Differences in the distribution patterns of the sand fly species as well as in species richness and abundance between the different ecotopes were investigated during both rainy and dry seasons over the course of the study period.

**Results:**

A total of 8,046 sand flies belonging to 11 genera and 28 species were collected. *Lutzomyia longipalpis* and *Nyssomyia intermedia* were the most abundant species in peridomicile areas, whereas *Martinsmyia minasensis* and *Lutzomyia cavernicola* were the most abundant species among the different trail ecotopes.

**Conclusion:**

The different composition of the sand fly fauna observed in the peridomicile areas and in the trails during the study, reinforces the importance of sampled different areas in a phlebotomine fauna survey. The presence of *Lutzomyia longipalpis* and *Ny. Intermedia* most abundant in peridomicile can be important to *Leishmania infantum* and *Leishmania braziliensis* transmission in the Imbaúbas native village.

## Background

Sand flies have been the subject of intense study, primarily in the context of the epidemiology of several diseases, the most notable being leishmaniasis [1]. In addition to *Leishmania*, sand flies also serve as hosts to bacteria, fungi, certain plasmodium species, haemogregarines, trypanosomes and *Endotrypanum *[2-6].

Although sand flies are distributed worldwide, they are most abundant in Neotropical regions, where a large number of species can be found [7]. In particular, there are approximately 900 species of sand flies worldwide, with over 500 in Neotropical regions and about 230 in Brazil [8,9]. In the state of Minas Gerais, about 100 species of sandflies have been reported, including important vectors of American Cutaneous Leishmaniasis (ACL) and Visceral Leishmaniasis (VL), such as *Bichromomyia flaviscutellata*, *Lutzomyia longipalpis*, *Migonemyia migonei*, *Nyssomyia intermedia* and *Nyssomyia whitmani *[10-14].

Cases of leishmaniasis within indigenous lands in Brazil were first reported in the state of Mato Grosso [15], who reported a large number of ACL cases among the Waurá Amerindians from Alto Xingu. In addition, the epidemiological profiles for VL among the Macuxí and Yanomami in the state of Roraima were described [16-18]. More recently, the prevalence of ACL in the Xakriabá Indigenous Reserve (XIR), state of Minas Gerais were reported [19]. Furthermore, the role of sandflies in the transmission cycle of *Leishmania* within indigenous land has been reported in the Brazilian states of Mato Grosso [20] and Mato Grosso do Sul [21].

Knowledge concerning the Phlebotominae fauna is essential to understanding the transmission of leishmaniasis. Therefore, the aim of this study was to evaluate the ecological parameters of the Phlebotominae fauna – focusing on potential *Leishmania* vectors – within the XIR, where numerous cases of cutaneous leishmaniasis have been reported.

## Methods

### Study area

**Figure 1:**
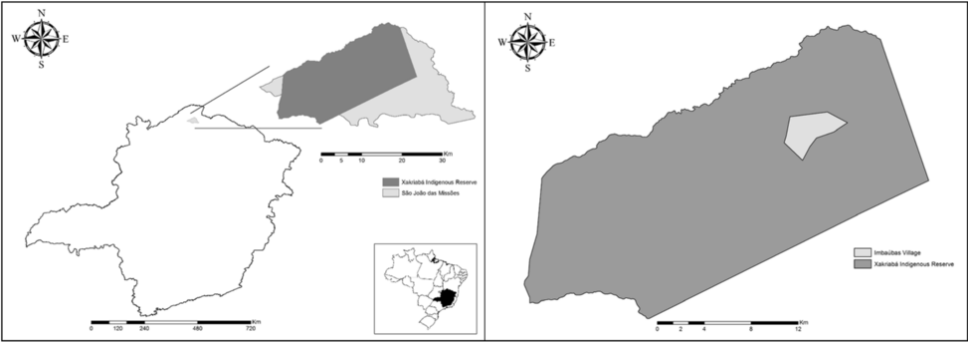
**Location of study area.** The location of the municipality of São João das Missões in the north of Minas Gerais state, Brazil. The Imbaúbas native village in the Xakriabá Indigenous Reserve, where the study was performed, is indicated.

The XIR is located in the municipality of São João das Missões (14°53′4.26“S 44°4′53.19”W) in the northern region of the state of Minas Gerais, Brazil (Figure 1). The indigenous reserve is located in a transition zone between the cerrado and the caatinga biomes and contains native species of both biomes. This study was conducted in the Imbaúbas native village, which had both a high prevalence of human leishmaniasis cases and a number of wild, synanthropic and domestic animal species known to carry *Leishmania *[22] and was conducted under authorization by FUNAI (National Indian Foundation – Protocol Number: 2098/08).

### Sand fly collection and environmental characteristics

**Figure 2:**
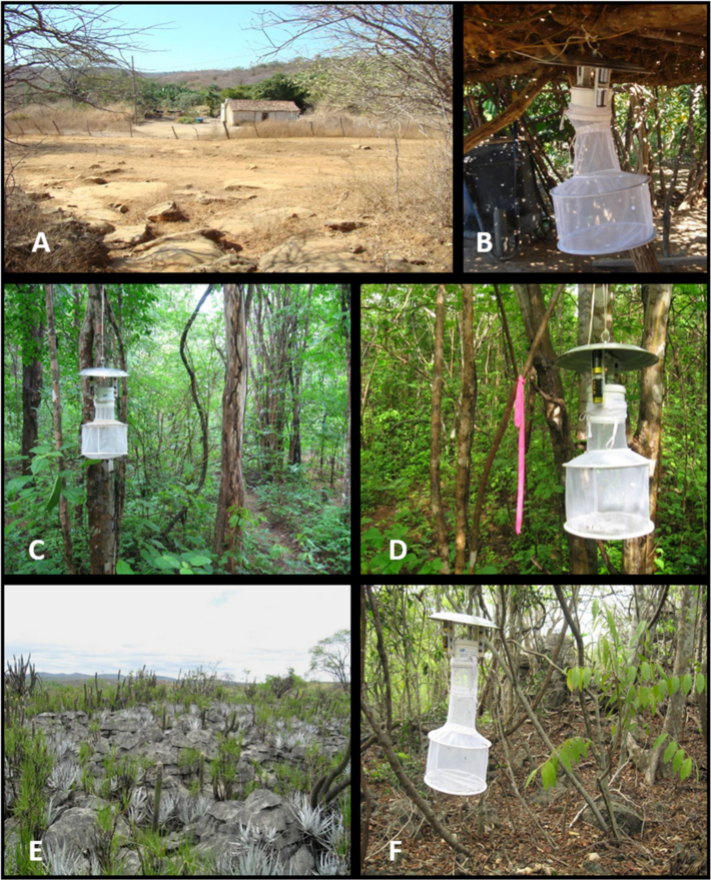
**Different ecotopes where the study was performed. A)** The peridomicile area of a household in the Imbaúbas native village of the Xakriabá Indigenous Reserve. **B)** An HP light trap set in the peridomicile area of a household. **C**, **D**, **E** and **F)** Representative images of trails 1, 2, 3 and 4, respectively.

Sand flies were captured with HP light traps [23] on three consecutive nights (from 6 pm to 6 am) for six months between July 2008 and July 2009. A total of 40 traps were installed in 20 randomly selected households in each of the sampled months. (Figure 2A and B).

To collect sand flies from the different ecotopes of the native village, bimonthly systematic samplings were performed between October 2011 and August 2012 on trails previously selected for the study of *Leishmania* reservoirs [22]. Four trails (330 meters in length each) were selected, and five traps were placed along each trail, totaling 20 HP traps. The traps were set for three full days, for a total of 72 hours of sampling per trap per collection.

Trails 1 and 2 (Figure 2C and D) cut through forest environments with little anthropization characterized as transitional between seasonal deciduous forest and cerrado. Along these two trails, climatic variations throughout the year can abruptly modify soil conditions and vegetation, potentially influencing sand fly abundance and richness. Trail 3 (Figure 2E) was situated on a plateau containing rocky outcrops with numerous craters, favorable for both sand flies and rodents. Trail 3 had a small amount of vegetation with caatinga characteristics. Trail 4 (Figure 2F) was located on the edge of a rocky outcrop plateau that could be characterized as a transitional region between cerrado forest and caatinga vegetation.

### Processing of collected sand flies

Preparation and mounting of the sand flies was performed using Canada balsam for males and Berlese’s medium for females [24]. Sand flies were identified to species level using microscopic observation of various internal and external morphological characteristics and using the keys and classification proposed by Galati [8]. The species abbreviations used in the present study follow the proposal by Marcondes [25].

### Climate data

Bioclimatic data, including relative humidity, rainfall and average temperature, were obtained from the National Institute of Meteorology for each month of the study period. Data were also collected at the Mocambinho Automatic Weather Station, which is located in the municipality of Itacarambi, state of Minas Gerais, approximately 30 km from the XIR [26].

Characterization of dry and rainy seasons was performed based on the rainfall of the months in which the captures were performed. During the years in which the peridomicile collections were performed, the dry season included the months of July 2008, September 2008 and July 2009, and the rainy season included November 2008, January 2009 and April 2009. For the collections performed on the trails, the dry season included the months of April 2012, June 2012 and August 2012, and the rainy season included October 2011, December 2011 and February 2012.

### Statistical analysis

Data meeting the prerequisite for normality were subjected to analysis of variance (ANOVA) (Kolmogorov-Smirnov and Lilliefors test, p < 0.05). Non-parametric data were analyzed using the Kruskal-Wallis test by rank and median. Richness and abundance data were considered as dependent variables, whereas season, month, location and sand fly sex were considered as categorical variables. All analyses were performed using the Statistica 10.0 software program (Statsoft, Tulsa, USA).

## Results

A total of 8,046 sand fly specimens from 11 genera and 28 species were collected in the XIR over the course of the study, 5,406 of which were female (67.2%) and 2,640 of which were male (32.8%). In the peridomicile areas, 2,126 (26.4%) sand fly specimens from 9 genera and 19 species were collected. We observed a significant difference between the number of males and females collected in peridomicile areas (H_1.198_ = 26.62; p = 0.000), with more females (1,385) than males (741) collected. The most commonly sampled species and their relative frequencies within peridomicile areas were *Lutzomyia longipalpis* (51.08%), *Nyssomyia intermedia* (31.79%) and *Micropygomyia goiana* (3.01%). Along the different trail ecotopes, 5,920 sand fly specimens (73.6% of total) from 11 genera and 27 species were collected. We also observed a significant difference between the numbers of males and females collected along the trails (F_1.36_ = 28.239; p = 0.000). The most commonly sampled species along the trail ecotopes were *Martinsmyia minasensis* (26.7%), *Lutzomyia cavernicola* (17.24%) and *Lutzomyia renei* (14.0%). The highest sand fly abundance was observed along trail 3 (52.58%), followed by trail 4 (34.71%), whereas only 12.71% of the total sand flies were collected along the other trails (p < 0.05). An analysis of species richness revealed a significant difference between the number of species collected on the different trails (F_3.20_ = 7.1566; p < 0.05), with trail 2 showing the fewest number of species (15 species). We sampled 20, 20 and 21 different sand fly species on trails 3, 4 and 1, respectively, with no significant differences in species richness between these trails.

Table 1 shows the distribution of the species collected in the peridomicile areas during the studied months. The months with the highest sand fly abundances were November 2008 (36.92%) and September 2008 (25.77%), with the other months accounting for 37.31% of the sand flies collected. We observed a significant difference in abundance (H_5.99_ = 18.44; p = 0.002) and species richness between the different months (H_5.99_ = 29.58; p = 0.000).

**Table 1:** Sand flies collected by sex and seasons in peridomicile located in Imbaúbas village, Xakriabá Indigenous Reserve, MG, from July 2008 to July 2009

	**Sampling months**														
	**Dry season**						**Rainy season**						**Total**		
**Species**	**Jul-08**		**Sep-08**		**Jul-09**		**Nov-08**		**Jan-09**		**Apr-09**				
	**♀**	**♂**	**♀**	**♂**	**♀**	**♂**	**♀**	**♂**	**♀**	**♂**	**♀**	**♂**	**♀ (%)**	**♂ (%)**	**Total (%)**
*Brumptomyia avellari*	0	1	2	3	1	2	15	14	0	0	1	0	19 (48.7)	20 (51.3)	39 (1.80)
*Evandromyia lenti*	3	4	10	7	1	1	13	6	0	0	1	0	31 (60.7)	20 (39.3)	51 (2.30)
*Evandromyia cortelezzii*	0	0	6	0	0	0	0	3	0	0	1	1	7 (63.6)	4 (36.4)	11 (0.50)
*Evandromyia cortelezzii* complex	0	0	0	0	0	0	20	0	2	0	0	0	22 (100)	0 (0)	22 (1.00)
*Evandromyia sallesi*	1	0	1	0	0	0	0	2	0	0	0	0	2 (50)	2 (50)	4 (0.10)
*Evandromyia spelunca*	0	0	0	1	1	0	4	0	0	0	0	0	5 (80)	1 (20)	6 (0.20)
*Evandromyia termitophila*	0	0	1	0	1	3	0	0	0	0	1	0	3 (50)	3 (50)	6 (0.20)
*Lutzomyia ischnacantha*	0	0	6	2	0	0	10	2	0	0	2	3	18 (72)	7 (28)	25 (1.10)
*Lutzomyia longipalpis*	6	39	176	60	33	42	260	295	40	54	27	54	542 (49.9)	544 (50.1)	1086 (51)
*Lutzomyia* sp.*	1	0	0	6	0	0	15	5	0	1	11	2	27 (65.8)	14 (34.2)	41 (1.90)
*Lutzomyia renei*	0	1	14	0	0	0	16	0	1	0	2	0	33 (97)	1 (3)	34 (1.60)
*Martinsmyia minasensis*	0	0	0	0	0	0	1	0	0	0	0	0	1 (100)	0 (0)	1 (0.04)
*Micropygomyia capixaba*	0	0	0	0	0	3	0	0	1	0	2	0	3 (50)	3 (50)	6 (0.20)
*Micropygomyia goiana*	0	2	10	2	1	1	24	19	2	0	2	1	39 (60.9)	25 (39.1)	64 (3)
*Micropygomyia peresi*	1	0	0	0	1	1	6	1	2	0	2	0	12 (85.7)	2 (14.3)	14 (0.60)
*Micropygomyia quinquefer*	0	0	0	0	0	0	0	0	2	0	0	0	2 (100)	0 (0)	2 (0.09)
*Migonemyia migonei*	1	5	1	7	2	1	3	5	1	1	0	0	8 (29.6)	19 (70.4)	27 (1.20)
*Nyssomyia intermedia*	179	15	209	20	87	18	40	4	8	3	82	11	605 (89.4)	71 (10.6)	676 (32.78)
*Nyssomyia whitmani*	0	0	2	1	0	0	0	0	0	1	1	0	3 (60)	2 (40)	5 (0.20)
*Pintomyia serrana*	0	0	1	0	0	0	2	0	0	0	0	1	3 (75)	1 (25)	4 (0.10)
*Sciopemyia sordellii*	0	0	0	0	0	0	0	0	0	2	0	0	0 (0)	2 (100)	2 (0.09)
**Total (%)**	**192 (74.1)**	**67 (25.9)**	**439 (80.1)**	**109 (19.9)**	**128 (64)**	**72 (36)**	**429 (54.6)**	**356 (45.4)**	**59 (48.7)**	**62 (51.3)**	**138 (64.7)**	**75 (35.3)**	**1385 (65.1)**	**741 (34.9)**	**2126 (100)**
	**259 (12.18)**		**548 (25.77)**		**200 (9.43)**		**785 (36.92)**		**121 (5.69)**		**213 (10.01)**		**2126**		

*The specimens have damaged essential morphological structures.

Species richness was significantly different between the dry and rainy seasons (H_1.99_ = 4.57; p = 0.0325), and a greater number of sand fly species was collected during the rainy season in peridomicile areas.

Distribution of sand fly species collected along the trails on a month-by-month basis is shown on Table 2. A significant difference in sand fly abundance was observed between the different months (F_5.12_ = 48.257; P = 0.000), with the highest abundances being observed in December 2011 (22.9%) and June 2012 (21.6%); there was no significant difference in abundance between these two months. However, we observed no significant difference in species richness based on month for the different trails (F_5.12_ = 1.5990; p = 0.23381).

**Table 2:** Distribution of sandfly species collected on tracks according to sex, sampling months and seasons in Imbaúbas village, Xakriabá Indigenous Reserve, MG

	**Sampling months**												**Total**		
**Species**	**Oct-11**		**Dec-11**		**Feb-12**		**Apr-12**		**Jun-12**		**Aug-12**				
	**♀**	**♂**	**♀**	**♂**	**♀**	**♂**	**♀**	**♂**	**♀**	**♂**	**♀**	**♂**	**♀ (%)**	**♂ (%)**	**Total (%)**
*Brumptomyia avellari*	1	1	0	0	0	0	1	0	3	1	0	0	5 (71.4)	2 (28.6)	7 (0.1)
*Brumptomyia brumpti*	0	0	0	0	0	0	0	0	2	0	0	0	2 (100)	0 (0)	2 (0.04)
*Evandromyia cortelezzii*	0	0	4	0	0	0	0	0	0	0	3	0	7 (100)	0 (0)	7 (0.1)
*Evandromyia evandroi*	0	0	0	0	0	0	0	0	7	0	0	0	7 (100)	0 (0)	7 (0.1)
*Evandromyia lenti*	6	10	0	7	6	5	4	18	66	25	4	3	86 (55.8)	68 (44.2)	154 (2.61)
*Evandromyia sallesi*			1	0	1	0	2	0	0	0	0	0	0	0	4 (100)
*Evandromyia* sp.*	0	4	0	0	0	0	0	0	0	0	0	0	0 (0)	4 (100)	4 (0.07)
*Evandromyia spelunca*	0	50	28	43	8	84	16	63	16	51	11	31	79 (19.7)	322 (80.3)	401 (6.82)
*Evandromyia termitophila*	4	10	0	1	0	0	1	2	0	13	2	3	7 (19.4)	29 (80.6)	36 (0.6)
*Lutzomyia cavernicola*	0	0	2	263	5	49	2	118	2	416	1	180	12 (1.1)	1026 (98.9)	1038 (17.52)
*Lutzomyia ischnacantha*	0	6	28	64	11	40	25	41	58	59	41	10	163 (42.5)	220 (57.50)	383 (6.50)
*Lutzomyia longipalpis*	7	4	0	1	0	2	3	5	29	28	2	1	41 (50)	41 (50)	82 (1.40)
*Lutzomyia renei*	13	5	107	28	59	0	85	134	270	0	131	0	665 (79.9)	167 (20.1)	832 (14)
*Lutzomyia* sp.*	0	39	0	0	0	0	0	0	0	0	0	0	0 (0)	39 (100)	39 (0.60)
*Martinsmyia minasensis*	58	559	150	234	98	341	18	64	6	37	2	0	332 (21.1)	1235 (78.9)	1567 (26.62)
*Micropygomyia capixaba*	0	0	12	114	0	45	0	48	0	0	2	0	14 (6.3)	207 (93.7)	221 (3.70)
*Micropygomyia goiana*	1	96	5	29	8	9	22	74	26	45	4	16	66 (19.7)	269 (80.3)	335 (5.70)
*Micropygomyia longipennis*	0	8	0	12	0	0	0	0	0	2	0	0	0 (0)	22 (100)	22 (0.30)
*Micropygomyia peresi*	27	37	152	34	7	33	84	57	32	10	12	0	314 (64.7)	171 (35.3)	485 (8.10)
*Micropygomyia quinquefer*	0	0	0	0	0	0	2	0	0	0	0	0	2 (100)	0 (0)	2 (0.04)
*Micropygomyia schreiberi*	0	0	21	0	3	0	2	2	4	10	4	35	34 (41.9)	47 (58.1)	81 (1.40)
*Micropygomyia* sp.*	0	12	0	0	0	0	0	0	0	1	0	0	0 (0)	13 (100)	13 (0.20)
*Migonemyia migonei*	0	2	0	0	0	1	0	0	1	0	0	0	1 (25)	3 (75)	4 (0.07)
*Nyssomyia intermedia*	1	9	5	6	9	42	14	42	17	39	0	1	46 (24.8)	139 (75.2)	185 (3.20)
*Nyssomyia neivai*	0	0	0	0	0	0	0	0	0	2	0	0	0 (0)	2 (100)	2 (0.04)
*Pintomyia misionensis*	0	0	0	0	0	0	0	1	0	0	0	0	0 (0)	1 (100)	1 (0.02)
*Pintomyia serrana*	0	0	0	0	0	0	0	1	0	0	0	0	0 (0)	1 (100)	1 (0.02)
*Psathyromyia (Foratiniella)* sp.*	0	0	0	0	0	1	0	0	0	0	0	0	0 (0)	1 (100)	1 (0.02)
*Psychodopygys ayrozai*	0	0	1	0	0	0	0	0	0	0	0	0	1 (100)	0 (0)	1 (0.02)
*Scyopemyia sordellii*	0	0	0	0	1	0	0	1	0	1	0	0	1 (33.4)	2 (66.6)	3 (0.06)
**Total (%)**	**119 (12.3)**	**852 (87.7)**	**516 (38.1)**	**836 (61.9)**	**217 (24.9)**	**652 (75.1)**	**279 (29.3)**	**671 (70.7)**	**539 (42.1)**	**740 (57.9)**	**219 (43.8)**	**280 (56.2)**	**1889 (31.9)**	**4031 (68.1)**	**5920 (100)**
	**971 (16.4)**		**1352 (22.9)**		**869 (14.7)**		**950 (16.0)**		**1279 (21.6)**		**499 (8.4)**		**5920**		

*The specimens have damaged essential morphological structures.

A significant difference in sand fly abundance was observed between the dry and rainy seasons when the collections were performed on the trails, with the rainy season showing the greatest number of sand flies (F_1.36_ = 4.7571; p = 0.03579).

Female sand flies were more abundant than males during both the dry and rainy seasons but were more predominant during the rainy season (F1.36 = 8.7494; p = 0.00544). However, there was no significant difference in the number of sampled species in terms of males or females based on season (F1.36 = 2.6521; p = 0.11214).

**Figure 3:**
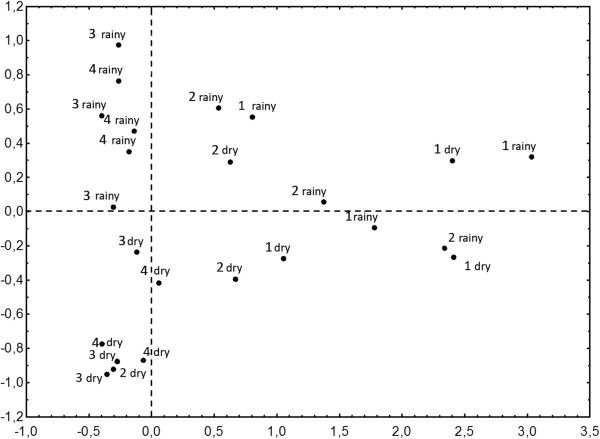
**Statistical analysis comparing the sand fly fauna.** A multivariate analysis comparing the Phlebotominae fauna between the different studied environments (forest, rocky outcrop, transition forest) and seasons (dry and rainy). Each point corresponds one month of collection in each trail, totaling six points per trail.

The results of the multivariate analysis comparing the compositions of the Phlebotominae fauna among the different studied environments (forest, rocky outcrop and transition forest) and seasons (dry and rainy) are shown in Figure 3. The vertical line passing through the X-axis origin shows the difference in the sand fly fauna compositions between the forest area (trails 1 and 2), grouped in the right side of the line, while the rocky outcrop (trail 3) and the transitional region (trail 4) grouped in the left side, show that the sand fly fauna composition in the study area was different. Furthermore, the horizontal line passing through the Y-axis origin highlights the difference in sand fly fauna composition between the rainy (above the line) and dry (bellow the line) seasons.

## Discussion

A number of studies have described the behavior of the Phlebotominae fauna, primarily those of medical importance [27-29]. Sand flies can be commonly found in a variety of natural ecotopes, including tree trunks, animal burrows, dead leaves, and rocky crevices [30-34], as well as in rural and urban environments close to domestic animal shelters and human residences [35-39]. Considering the diversity of ecotopes in which sand flies can be found, sampling from a variety of different environments within a given study area is crucial for fauna studies. In this study, we collected sand flies from several different areas, including peridomicile areas around households as well as trails containing a variety of ecotopes, such as cerrado forests, seasonal deciduous forests, cerrado *strictu sensu* and transition areas between cerrado and caatinga.

A great variety of genera and species were found in the Imbaúbas native village, corresponding to about 30% of the total registered species in the state of Minas Gerais [10-14].

Despite the fact that the collections in the peridomicile areas and along the trails were performed during different periods, precluding a statistical comparison between the ecotopes, certain findings were still significant. With respect to species richness, a higher diversity of sand flies was observed in the trails when compared to peridomicile areas. This difference was likely due to the diversity of ecotopes found along the trails as well as the preferences of the sand flies for the specific wild animals, domestic animals or humans found in these areas. By definition, peridomicile areas are significantly impacted by human intervention, which likely hinders the adaptation of certain sand fly species to these areas. It is important to note that the peridomicile areas surrounding houses within the XIR possess numerous rural characteristics, such as domestic animals, fruit trees and grain plantations, that provide shelter and food sources for adult sand flies as well as organic matter for the development of immature stages. Therefore, these characteristics may explain the significant number of species (19) found in this ecotope.

In the peridomicile areas, *Lu. longipalpis* and *Ny. intermedia* were the predominant species, which was significantly different from what was observed along the trails, where these species were relatively rare*.* Importantly, these species are involved in transmission of the VL and ACL etiological agents in several endemic areas of Brazil [1,40].

Since the VL transmission cycle began to be elucidated in Brazil in the 1930s, several research groups have demonstrated the ability of *Lu. longipalpis* to adapt to human-modified environments as well as its crucial role in the transmission of *Leishmania infantum *[1]. Therefore, the near constant presence of *Lu. longipalpis* in peridomicile, where it can feed on domestic and synanthropic hosts of *Leishmania*, associated with their anthropophily contribute to its vectorial capacity [41,42]. As a consequence, *Lu. longipalpis* plays an important role in the transmission of VL in peridomicile areas of both rural and urban regions [41,43,44].

Our results agree with previous entomological studies performed in the north of Minas Gerais state. In these studies, *Lu. longipalpis* was identified as the predominant species found in peridomicile areas [45,46]. Correlations between *Lu. longipalpis* density and specific conditions in this environment have been noted, and this species is commonly associated with the presence of domestic animals [47-50]. This behavioral trait was also observed in the present study, as we found domestic animals, including chickens, pigs and dogs in the peridomicile areas.

With respect to *Ny. intermedia*, most of the specimens were collected in peridomicile areas and few specimens were collected in forest fragments near residences. These results are consistent with the reports of Forattini in the Paulista plateau, state of São Paulo [47,51] and Rangel *et al*., in the municipality of Mesquita, state of Rio de Janeiro [52]. These authors showed that *Ny. intermedia sensu lato* lives in close association with humans as well as domestic and synanthropic animals in a variety of habitats, including peridomicile and forested areas. Epidemiological evidence accumulated over the years suggests that *Ny. intermedia sensu lato* is the primary vector of the ACL etiological agent in endemic areas of southeastern Brazil [53-57], and the distribution of this species consistently coincides with the distribution of ACL in humans [51,58-61]. Therefore, based on the proven epidemiological importance of *Ny. intermedia sensu lato* and its high population density within peridomicile areas, this species likely plays an important role in the *Leishmania* transmission cycle in the XIR.

Despite being found in low numbers in the present study, the presence of the species *Ev. cortellezii*, *Ev. sallesi, Ev. termitophila, Migonemyia migonei, Ny. neivai* and *Ny. whitmani* should also be addressed. These species have been found to be naturally infected by *Leishmania* in Brazil, thus implicating them as potential etiological vectors, and some may be sporadically involved in *Leishmania* transmission [40,56,62,63].

It is necessary to highlight the presence of *Ev. lenti* in both the peridomicile and trail areas, despite the small number of collected specimes. In a study performed in Ceará state – Brazil [64], although only a small number of *Ev. lenti* individuals were found in peridomicile areas, a large number were found inside the associated households. In the municipality of Jacobina, state of Bahia - Brazil, *Ev. lenti* specimens that were naturally infected with promastigotes were found biting humans, horses and dogs [65]. In contrast with these reports, in a study of *Ev. lenti* biology involving a population from the state of Minas Gerais, demonstrated that this species does not have anthropophilic habits and is refractory to *Leishmania* species [66]. However, recently, cases of natural infection with *Le. braziliensis* by *Ev. lenti* were reported and verified using molecular biology techniques [67,68]. Therefore, the epidemiological role of *Ev. lenti* must be clarified, which is highlighted by the fact that this species was collected in both peridomicile and forested areas near humans in this study.

Regarding the Phlebotominae fauna collected along the trails, it was observed that trail 3 showed the highest sandfly abundance. During the study period, about 40% of the collected specimens were captured in this ecotope. Furthermore, a high species richness was observed. This phenomenon was primarily due to the environmental composition of trail 3, which consisted of vertical rocky outcrops that formed craters in the soil where temperature and relative humidity remained relatively stable throughout the day, making it a suitable habitat for the breeding and establishment of a variety of vertebrate species, including small- and medium-sized rodents and bats that can serve as food sources for sand flies.

The presence of *Mt. minasensis* was largely confined to trails 3 and 4 and little is known concerning the feeding behaviors and habits of this species. Species of the genera *Martinsmyia* can be attracted by rodents, as suggested to *Mt. gasparviannai* in the state of Espírito Santo [69] and *Mt. oliveirai* in the state of Mato Grosso do Sul [34]. In addition to the high prevalence of *Mt. minasensis* along the trails, the regular presence of *Mi. peresi, Mi. goiana, Lu. renei, Lu. cavernicola* and *Ev. spelunca* throughout the year suggests that these species use these wild environments as breeding sites, as specimens of both sexes were routinely collected. For the remaining species that were only sporadically collected, our findings suggest that these trails serve only as temporary shelters [70].

The impact of climatic factors on sand fly populations has been addressed by several authors. According to the literature, temperature, humidity and rainfall can influence sand fly populations in varying ways, depending on the region studied. Rutledge & Ellenwood [71] suggest that sand fly seasonality is related to rainfall distribution patterns, which affect breeding conditions on the ground. In our study, we observed significant correlations between sand fly abundance and the rainy season along the trails, as well as for species richness and the rainy season in the peridomicile areas. Indeed, significant correlations between season and fluctuations in Phlebotominae populations have been observed in several Brazilian states, including Minas Gerais [46,72], Rio Grande do Norte [73], Mato Grosso [74], Mato Grosso do Sul [75,76], Ceará [41] and Bahia [48].

Even slight variations in certain climatic factors can affect sand fly micro-habitat enough to alter population dynamics, as these insects are very sensitive to desiccation [45]. Therefore, this phenomenon may explain the higher number of sand flies collected along trail 3, which consisted of rocky outcrops that could serve as micro-habitat with relatively stable temperature and humidity throughout the year. However, in the forest, transition and peridomicile areas, climatic changes occur more frequently, leading to environmental changes throughout the year. For example, during the driest months, reduced rainfall levels result in drastically reduced vegetation cover (characteristic of seasonal deciduous forests), which may directly affect the sand flies breeding sites.

## Conclusion

In recent decades, significant changes have been occurring in many natural environments, mostly due to human activity. As a result, many insects that transmit disease and various components of parasitic life cycles are beginning to show population dynamics and interactions different from their original descriptions. Therefore, studies involving fauna surveys that also address biological aspects of specific vectors contribute to a better understanding of the dynamic interactive processes between hosts and parasites. This study addressed the sand fly fauna in a significant leishmaniasis endemic area, and these results will enhance our knowledge concerning the number of sand fly species found in this region as well as the distribution of these species across different ecotopes. This knowledge may prove useful for establishing more effective prophylactic measures.

## Abbreviations

XIR: Xakriabá indigenous reserve; ACL: Cutaneous Leishmaniasis; VL: Visceral Leishmaniasis; FUNAI: National Indian Foundation.

## Competing interests

The authors declare that they have no competing interests.

## Authors’ contributions

FDR: designed the study, field work, processed and identified the collected sand flies and wrote the manuscript. PHFS: field work, identified the collected sand flies and reviewed the manuscript; PFQ: designed the study, field work and statistical analysis; IRC: statistical analysis; GBT: field work and processed the collected sand flies; KMSS: field work and processed the collected sand flies; RAB: processed the collected sand flies, conceived and designed the experiments; ESD: conceived and designed the experiments; CMFG: designed the study, financial support for the study and reviewed the manuscript. All authors approved the final version of the manuscript.
